# Genotoxic and cytotoxic effects of cone beam computed tomography on exfoliated epithelial cells in different age groups

**DOI:** 10.1186/s12903-025-06422-3

**Published:** 2025-07-09

**Authors:** Maged Bakr, Fatma Ata, Asmaa Saleh Elmahdy, Bassant Mowafey

**Affiliations:** 1 Department of Oral Medicine, Periodontology, Diagnosis and Oral Radiology, Faculty of Dentistry, Mansoura University, El- gomhouria Street, P.O. Box: 35516, Mansoura City, Dakahlia, Egypt; 2 Department of Oral Biology Faculty of Dentistry, Mansoura University, 60 El-gomhouria Street, P.O. Box: 35516, Mansoura City, Dakahlia, Egypt

**Keywords:** Micronucleus, Cone beam computed tomography, Cytological changes, Buccal mucosa

## Abstract

**Background:**

Radiographic examination is an essential method for dental practitioners. Cone beam computed tomography (CBCT) is considered a common radiographic technique for dental problems. However, there are serious concerns regarding the biological effects of radiation, particularly at a young age. The objective of the present study was to evaluate the cytotoxic and genotoxic effects of cone beam computed tomography on the exfoliated buccal epithelial cells in two distinct age groups.

**Methods:**

Forty patients were included in the current study. They were divided into two groups: group I included patients ≥ 18 years old, and group II included those ˂18 years old. They experienced CBCT as part of a diagnostic procedure. Cells were collected before CBCT exposure and 10–12 days after CBCT exposure. The Papanicolaou method was utilized to stain cytological smears. Micronuclei count and cytotoxic cellular alterations (Karyolysis, Pyknosis, and Karyorrhexis) were assessed. The Mann-Whitney U test was used to compare the two studied groups for non-normally distributed data. A two-way ANOVA test was used to study the combined effect of two independent factors on dependent continuous outcomes.

**Results:**

In groups I and II, there were statistically significant differences in micronucleus counts and cytological changes before and after CBCT exposure. There were no statistically significant differences between the two groups in micronucleus counts and cytological changes after CBCT exposure.

**Conclusions:**

CBCT induces genotoxic and cytotoxic effects in both age groups, with cytotoxicity more evident in younger patients. Therefore, clinical justification is required before using CBCT.

## Background

Radiographic examination plays a crucial role in diagnosing many diseases, predicting prognosis, and monitoring the treatment progress [1]. CBCT is a common technique in maxillofacial imaging. It offers numerous advantages, including rapid scanning duration and overlap-free reconstructed data for further applications such as volumetric analysis [2, 3]. Despite these advantages, ionizing radiation produced by CBCT can cause biological harm [4, 5]. 

There are two fundamental categories of radiation-induced biological effects: stochastic and deterministic. The distinguishing difference between these two categories is the dose threshold for their occurrence. Stochastic effects do not exhibit a dose threshold, whereas deterministic effects occur only when the radiation dose exceeds a certain threshold. Diagnostic radiation doses expose the patient to the risk of stochastic effects but not deterministic effects [6]. 

Children’s quickly developing tissues are considered more radiosensitive than the mature tissues of adolescents and adults, rendering them more vulnerable to radiation exposure. The child has a greater life expectancy than an adult; hence, the cumulative effects of radiation have a prolonged duration to induce cancer [7]. Additionally, it has been established that xenobiotic metabolizing enzyme systems and DNA repair systems are not fully mature in children [8]. 

A significant reduction in dosage is attained by choosing a field of view (FOV) that aligns with the region of interest [9, 10]. Selecting an appropriate FOV is essential to balance diagnostic efficacy with patient safety [11], A small field of view, restricted to a single tooth or quadrant, is indicated for diagnostic and therapeutic purposes in endodontics [12] A medium field of view is indicated in implant planning. The large field of view is recommended for specific cases including skeletal anomalies, orthodontic and orthognathic surgery [13]. 

It was reported that even a low dose of ionization radiation has the potential for cytotoxic effects and DNA damage, so clinical justification is required before using CBCT [14]. 

There are several ways to measure and analyze genomic damage. One of the most widely used and accurate tests is the micronucleus test [15]. The micronucleus is a tiny extranuclear body that forms during cell division due to aberrant chromosomes or incorrect mitotic spindle function [16]. As a result, the micronucleus assay is a widely used method for tracking people’s recent exposure to genotoxic substances like chemicals and ionizing radiation. This test is performed on human peripheral lymphocytes to identify genotoxic damage [14]. 

Since using exfoliated buccal epithelial cells is a cheap, noninvasive, and patient-motivated approach, it is preferable to use buccal epithelial cells rather than lymphocytes. Apart from the micronucleus test, many cytological changes, such as changes in chromatin status and nuclear abnormalities, have been identified in exfoliated buccal cells as markers of cytotoxic effects [14, 17]. 

This study evaluated the cytotoxic and genotoxic effects of cone beam computed tomography on the exfoliated buccal epithelial cells before and after 10–12 days of exposure in two distinct age groups.

## Methods

### Study design

This study assessed individuals at two different intervals, baseline and 10–12 days, following CBCT exposure. The study was approved by Mansoura University, Faculty of Dentistry, Research Ethics Committee with ethical permission number (NO A04030230M). Informed written consent was obtained from selected patients over 18 years old. For the patients under 18, the parents of the participating children provided informed written consent following a detailed explanation of the study procedures. The research was carried out following the ethical guidelines established by the Helsinki Declaration of 1964 related to human studies [18, 19]. 

### Sample

Sample size calculation was based on the mean percentage of cells with genotoxic changes (micronucleus) among the studied groups before and after 10–12 days of CBCT exposure, retrieved from previous research [20]. Using G*power [21] version 3.1.9.4 to calculate sample size based on effect size of 1.158, 2-tailed test, α error = 0.05, and power = 90.0%, the total sample size was 17 in each group at least. The dropout was estimated to be 15%, so the adjusted sample size was increased to 20 participants in order to compensate for any dropouts.

### Patients’ selection

The study was performed on 40 patients selected from the Faculty of Dentistry, Mansoura University outpatient clinic. These patients needed CBCT imaging for diagnostic purposes, including implant assessment and placement, third molar extraction, orthodontic treatment, and localized assessment of impacted and supernumerary teeth.

### Exclusion criteria

Patients exposed to CBCT one month before the study or with a previous history of taking radiotherapy and chemotherapy were not allowed to participate. In addition, patients with oral cancer or those having a red, white, or pigmented lesion were not included. Alcohol or tobacco consumers were also excluded. Individuals with systemic conditions that influence cytological outcomes, such as diabetes, autoimmune diseases, active viral infections, or chronic medication use that might alter cellular responses, were excluded.

### Patient grouping

The selected patients included in this study were divided into two groups:

**Group I** comprised 20 patients ≥ 18 years old.

**Group II** comprised 20 patients > 18years old.

### CBCT imaging protocol

Patients were scanned using a CBCT imaging machine (SCANORA^®^ 3D) operated at 84 kilovoltages, 9–14 milliamperage (mA), the medium field of view (FOV) 80 × 100 mm, and the exposure time was 6 s. The effective dose of the lower to mid-range of the reported values for medium FOVs was potentially between 50 and 200 µSv.

### Buccal mucosal cell collection and slide preparation

Exfoliated buccal cells were collected immediately before CBCT imaging and after 10–12 days after imaging. No specific dietary restrictions or instructions were provided between the CBCT imaging and cytological sample collection.

Each patient was instructed to rinse with tap water to eliminate any debris from the oral cavity. Exfoliated oral epithelial cells were taken by scraping the buccal mucosa with a moist wooden tongue depressor [22]. The pressure applied by the hand during smear preparation was sufficient to isolate only the surface epithelial cells of the mouth, and no bleeding occurred.

**Fig. 1:**
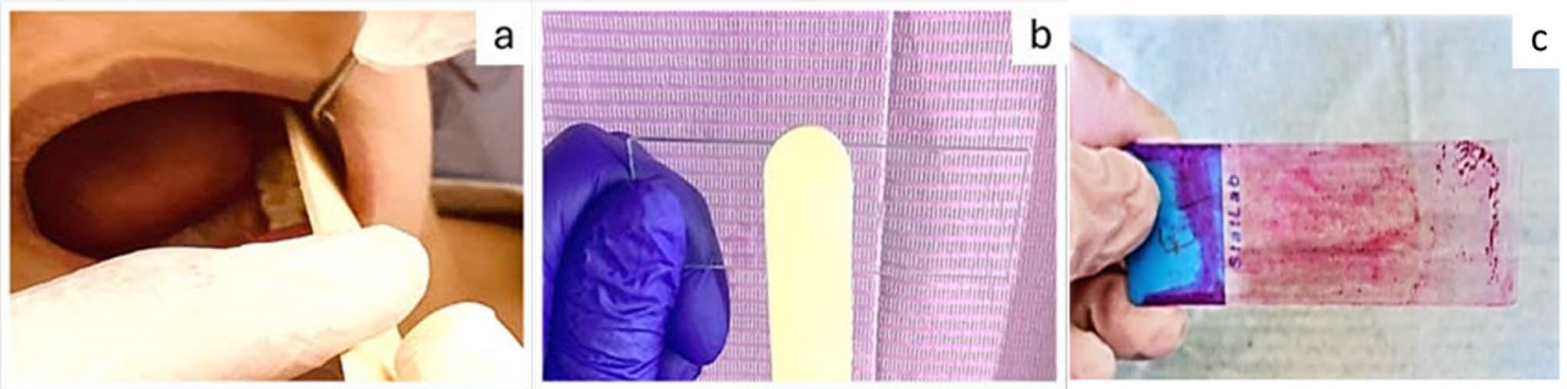
Method of obtaining buccal cytological smear, collection of buccal mucosal cells using a tongue depressor (**a**), smearing the obtained cells on a sterile glass slide (**b**), and a Papanicolaou-stained slide (**c**)

The obtained cells were smeared on a sterile glass slide and distributed over an extensive area, preventing the clumping of cells. Three slides for each patient were prepared. They were collected from both sides to increase the number of collected cells and to avoid bias, as sampling from both sides provides an overall view of cytotoxic and genotoxic damage. The smears were then fixed with 95% ethanol and stained using Papanicolaou stain, a polychromatic staining method used in cytopathology and histology that employs numerous dyes to stain different cell components differently. **(**Fig. 1**)**

### Cytological analysis and scoring

Using an MVV5000CL digital eyepiece fitted on a MEIJI MX5200L microscope, a 0.5 photo adaptor, and Future WinJoe software, 400X magnifications were achieved when photographing Pap-stained cytological smear slides. Micronucleus and other nuclear alterations were assessed in 1000 cells across two-time intervals for each patient. A blind inspection of each slide was performed by an oral biologist who was unaware of the patients’ identities or the time the cells were collected. To practice getting the sample, preparing the smear on slides, and counting the cells, a pilot study was conducted prior to the beginning.

### Assessment of genotoxicity and cytotoxicity

Each slide was evaluated for:

#### Micronucleus counts (a genotoxicity indicator)

Tolbert et al.‘s micronucleus identification parameters were applied while counting the micronuclei. It is a round, membranous extranuclear body that is one-third the size of the related nucleus on the same focal plane and has a staining intensity comparable to the nucleus [23]. 

#### Cytological changes (cytotoxicity indicators)

Nuclear alterations and various chromatin states, such as binucleation, broken egg nucleus, pyknosis (shrunken nuclei with uniformly stained nuclear material), condensed chromatin (aggregated chromatin regions - speckled nuclear pattern), karyorrhexis (more extensive condensed chromatin regions - nuclear fragmentation), and karyolysis (complete absence of DNA - ghost cell appearance), were investigated concerning cytological changes [23]. 

### Statistical analysis

Version 26 of the SPSS program (SPSS Inc., PASW Statistics for Windows) was used to analyze the data: SPSS Inc., Chicago. For non-normally distributed data, the median (lowest and maximum) and mean ± were used to characterize the quantitative data, and the standard deviation for data normally distributed after the Shapiro-Wilk test for normalcy. The acquired results were deemed significant at the 0.05 level.

The Mann-Whitney U test was used to compare the two studied groups for non-normally distributed data. A two-way ANOVA test was performed to study the combined effect of two independent factors on dependent continuous outcomes, with an estimation of R^2^, after the log transformation of the outcome to meet the assumptions of the parametric test.

## Results

The current study’s findings showed that the mean age of group I was 35.30 ± 10.26 years, whereas the mean age of group II was 14.40 ± 2.58 years. In group I, the male percentage was 45%, whereas in group II, it was 60%. 55% of females were identified in group I, whereas 40% were in group II. No statistically significant variation regarding sex was seen among the studied groups. (Table 1)

**Table 1:** Comparison of demographic characteristics between studied groups

	Group I *N* = 20(%)	Group II *N* = 20(%)	Test of significance
**Age/years Mean ± SD**	35.30 ± 10.26	14.40 ± 2.58	
**Sex**			
**Male**	9(45.0)	12(60.0)	X^2^ = 0.902 *P* = 0.342
**Female**	11(55.0)	8(40.0)	

X^2^=Chi-Square test

Group I patients ≥ 18 years old

Group II patients > 18years old

**Fig. 2:**
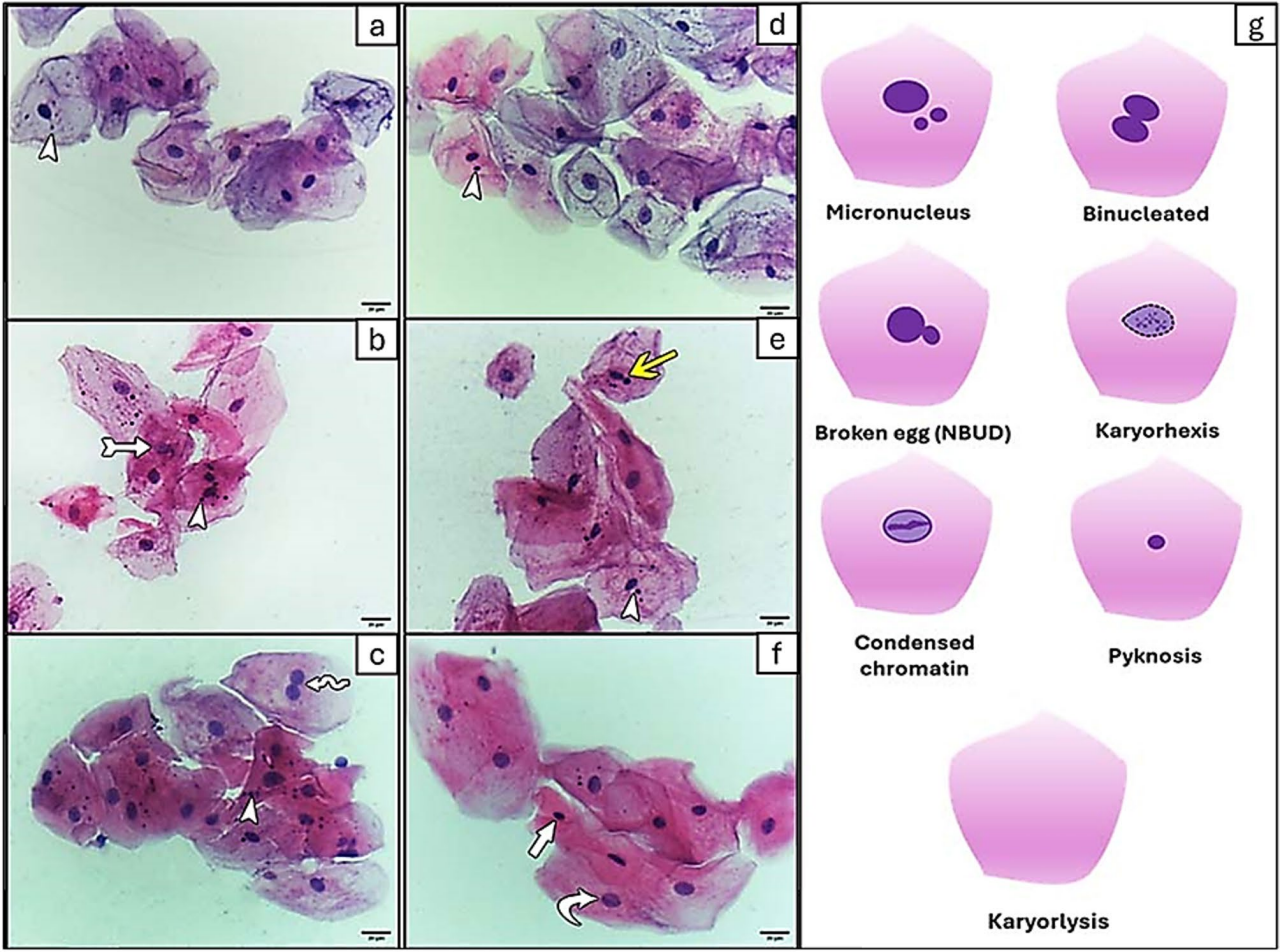
Photomicrographs of exfoliated buccal cells (Papanicolaou stain, 400X magnification, Bar = 20 μm) showing micronucleus and cytological changes before and after CBCT exposure in group I (**a-c**) and group II (**d-f**). Note: micronucleus (arrowhead), binucleated (wavy arrow), broken egg (yellow arrow), karyorrhexis (tailed arrow), condensed chromatin (curved arrow), and pyknosis (white arrow). Diagrammatic representations of different types of genetic and cytological damage that can be observed (**g**)

In group I, the micronucleus counts significantly increased from 9.1 ± 2.71 before CBCT exposure to 20.9 ± 4.18 10–12 days after exposure (*p* < 0.0001), reflecting a percentage change of 129.6%. Cytological changes exhibited a significant rise from 10.4 ± 4.88 before exposure to 21.3 ± 5.16 post-exposure (*p* < 0.0001), reflecting a percentage change of 104.8%. (Table 2; Fig. 2) In group II, elevations in micronucleus count and cytological alterations were seen, increasing from 8.3 ± 3.51 and 6.1 ± 3.97 before CBCT exposure to 22.7 ± 4.65 and19.3 ± 4.27 (*p* < 0.001) after exposure, (*p* < 0.001), reflecting percentage changes of 173.4% and 216.3%, respectively. (Table 2; Fig. 2) The Mann-Whitney U test revealed no statistically significant difference in micronucleus count before (P1 = 0.425) and after CBCT exposure (P2 = 0.205) between the two groups; the count of cytological changes was substantially lower in Group II compared to Group I before CBCT exposure (P1 = 0.001*). However, after exposure, the cytological changes in group II increased and became comparable to those in group I, with no statistically significant difference observed between the two groups (P2 = 0.189). (Table 2)

**Table 2:** Comparison of micronucleus counts and cytological changes before and after CBCT exposure

	Group I			Group II			Group I vs. Group II
	Before exposure	After exposure	Percentage of change	Before exposure	After exposure	Percentage of change	
Micronucleus count/1000 cells	9.1 ± 2.71 9(4–14)	20.9 ± 4.18 20(14–30)	129.6%.	8.3 ± 3.51 8(2–14)	22.7 ± 4.65 23(14–30)	173.4%	P1 = 0.425 P2 = 0.205
P 95%CI of difference	*P* < 0.0001* 8.82–14.78			*p* < 0.0001* 11.42–17.37			
Cytological changes count/1000 cells	10.4 ± 4.88 10(4–22)	21.3 ± 5.16 20(14–30)	104.8%.	6.1 ± 3.97 4(2–16)	19.3 ± 4.27 19(14–28)	216.3%	P1 = 0.001* P2 = 0.189
P 95%CI of difference	*P* < 0.0001* 6.64–15.15			*p* < 0.0001* 9.71–16.69			

Data expressed as mean ± SD, median (min-max),

P = p-value for comparing before and after CBCT exposure in the same group,

P1 = p-value for comparing group I and group II before CBCT exposure,

P2 = p-value for comparing group I and group II after CBCT exposure,

Used test: Mann-Whitney U test

*= Statistically significant at *p* < 0.05

CI: Confidence interval

Regarding the gender impact on outcomes, across both groups, no statistically significant differences were observed in the mean percentages of micronucleus counts or cytological changes between female and male participants, either prior to or following CBCT exposure. (Table 3).

**Table 3:** Comparison of micronucleus counts and cytological changes between females and males

		Group I		*P* value	Group II		*P* value
		Female	Male		Female	Male	
		Mean ± SD	Mean ± SD		Mean ± SD	Mean ± SD	
**Micronucleus counts**	**Before CBCT**	8.909 ± 3.270	9.333 ± 2.000	0.738	8.250 ± 2.493	8.333 ± 4.163	0.960
	**After CBCT**	20.727 ± 2.778	21.111 ± 4.125	0.844	24.750 ± 3.845	21.333 ± 4.774	0.109
**Cytological changes**	**Before CBCT**	8.727 ± 3.927	12.444 ± 5.365	0.090	5.750 ± 3.284	6.333 ± 4.499	0.757
	**After CBCT**	16.727 ± 2.412	16.222 ± 0.667	0.552	20.000 ± 4.536	18.833 ± 4.218	0.564

P-value comparing females and males before and after CBCT exposure in the same group

Data expressed as mean ± SD,

The two-way ANOVA results showed that age group and CBCT exposure were statistically significant determinants of cytological changes. The age group and CBCT exposure influenced 64.8% of cytological alterations, with a more pronounced effect observed for CBCT exposure (effect size 0.644) compared to the age group (effect size 0.110) (Table 4). The micronucleus count results revealed that CBCT exposure was the statistically significant predictor, with 74.7% of micronucleus count affected by CBCT exposure (effect size 0.755) (Table 4).

**Table 4:** Comparison of the combined effect of change in group and CBCT exposure on cytological changes and micronucleus count

	Cytological changes						Micronucleus Count					
Source	Type III Sum of Squares	Df	Mean Square	F	*P* value	Partial Eta Squared	Type III Sum of Squares	Df	Mean Square	F	*P* value	Partial Eta Squared
Corrected Model	3128.950^a^	3	1042.983	49.387	0.001*	0.661	3471.000^a^	3	1157.000	78.792	0.001*	0.757
Intercept	16302.050	1	16302.050	771.935	0.001*	0.910	18605.000	1	18605.000	1267.007	0.001*	0.943
Group	198.450	1	198.450	9.397	0.003*	0.110	5.000	1	5.000	0.341	0.561	0.004
CBCT exposure	2904.050	1	2904.050	137.513	0.001*	0.644	3432.200	1	3432.200	233.734	0.001*	0.755
Group * CBCT exposure	26.450	1	26.450	1.252	0.267	0.016	33.800	1	33.800	2.302	0.133	0.029
Error	1605.000	76	21.118				1116.000	76	14.684			
Total	21036.000	80					23192.000	80				
Corrected Total	4733.950	79					4587.000	79				
	**a. R Squared = 0.661 (Adjusted R Squared = 0.648)**						**a. R Squared = 0.757 (Adjusted R Squared = 0.747)**					

Test used: two-way ANOVA test (after log treansformation of cytological changes and micronucleus count to be continous normally distributed). The larger the effect size the stronger the relationship between two variables. A small effect size is typically considered to be around 0.01, a medium effect is around 0.06, and a large effect is around 0.14

Group (group I aged ≥ 18 years old, group II ˂ 18 years old)

## Discussion

Digital volume tomography, or CBCT, is increasingly used for maxillofacial imaging [24]. It has reduced radiation doses relative to conventional CT. However, it emits more radiation than standard dental X-rays, prompting concerns among dentists and patients, and may induce both gene mutations and chromosomal aberrations [25]. 

Assessing the genotoxic effects of X-rays is crucial due to substantial evidence correlating genetic damage with cancer development. Roughly 90% of oral malignancies in humans are thought to have their origins in the epithelium, making oral cancer the sixth most frequent cancer worldwide [26]. These facts highlight the importance of the micronucleus test, which shows the direct impact of harmful substances on target tissues like the buccal mucosa [27]. Many researchers were concerned about the radiation dose from CBCT and how it would affect living cells [28]. The present study assessed the cytotoxic and genotoxic effects of CBCT on the exfoliated buccal epithelial cells in two different age groups.

Micronucleus assays (MN) are classified into several in vivo/in vitro types, such as the cytokinesis-block micronucleus assay (CBMN), mammalian erythrocyte MN, buccal MN, and MN in other cell types. The frequency of micronuclei is the basis for each kind of MN, but they differ in the obtained cell type and methodology. MN has several advantages, including biomonitoring, genotoxicity, and cytotoxicity testing [29]. 

In the present study, buccal cells were obtained for micronuclei and other cytological evaluations since they are exposed to direct radiation during CBCT examination, rather than peripheral lymphocytes. This buccal MN assay allows for additional benefits such as ease of analysis, low cost, noninvasiveness, high accuracy, and speed [30]. The gingiva and dorsum surface of the tongue are also occasionally sampled. Still, they are difficult to stain due to their keratinization [31]. 

Genotoxic damage occurs in the basal cell layer of the epithelium, where cells go through mitosis, and can result in micronuclei formation. The rapid epithelium turnover causes these basal cells to migrate to the surface layer and undergo exfoliation [32]. Epithelial cells need approximately 7–16 days to reach the surface layer and then exfoliate [33]. Therefore, exfoliated buccal mucosa cells in the current study were collected immediately prior to exposure to ionizing radiation and 10–12 days after it.

The typical prevalence of cells with a micronucleus in the general population is 0.0-0.9%. Chromosome alterations may be the cause of any variation in MN. Cells that have sustained genetic damage have developed a micronucleus. It has long been thought that an increase in MN is associated with a rise in carcinogenic effects [34]. As the frequency of MN in the exfoliated cells of oral mucosa is low, the larger the count is, the more accurate the results are. Subsequently, at least 1000 normally differentiated cells must be evaluated at ×400 magnification to asses DNA damage biomarkers as suggested by Tolbert and Sarto [23, 33]. 

Micronuclei were analyzed using the standards provided by Sarto et al. and Tolbert et al. [26, 36]. Pyknosis, karyolysis, and karyorrhexis are examples of cytotoxic effects that have been examined since including them in assessments makes biomonitoring studies more sensitive [23]. 

The study findings indicated a significant increase in micronucleus count in group I, with age ≥ 18 years old, from 9.1 ± 2.71 before CBCT exposure to 20.9 ± 4.18 after exposure (*p* < 0.001), and in group II with age < 18 years old from 8.3 ± 3.51 before CBCT exposure to 22.7 ± 4.65, after CBCT exposure (*p* < 0.001). Regarding whether CBCT can harm cells genetically, the experimental results are controversial. The incidence of MN in oral exfoliated cells increased dramatically 10 days post-exposure to CBCT, as evidenced by. Basha [22], Altoukhi [20], Mosavat [35]. Additionally, this finding aligns with the results of the study by Fonte et al., conducted on adults to investigate the genotoxicity and cytotoxicity of CBCT in oral exfoliated cells confirming that CBCT poses a risk of inducing genetic damage [36]. However, Carlin [37], Lorenzoni [38], and Yang [31] could not identify a statistically significant change in MN frequency subsequent to exposure.

Regarding cytotoxicity, the cytological changes increase in group I from 10.4 ± 4.88 before exposure to 21.3 ± 5.16 (*p* < 0.001) and in group II from 6.1 ± 3.97 before CBCT to 19.3 ± 4.27. Previous studies have discovered various forms of cytotoxic changes in the oral mucosa cells of patients who had CBCT following a 10-day exposure. However, gender has no relation to the significance of CBCT genotoxicity and cytotoxicity side effects in the buccal mucosa [20, 22, 39]. 

In accordance with the earlier studies, the present study demonstrated no statistically significant gender differences in the average percentages of micronucleus counts or cytological alterations across all groups, regardless of the timing of CBCT exposure, which may contribute to the small size of the study groups.

When the two groups’ results were compared, the mean micronucleus count did not change significantly with age, which is consistent with the findings of Basha’s study [22]. 

Meanwhile, cytological alterations in children < 18 showed a significant decrease prior to CBCT exposure. However, after exposure, the cytological changes remarkably increased and became comparable to those of adults ≥ 18. This suggests that the cytotoxic effect of CBCT radiation is more evident in children than in adults, necessitating further research into whether it is a temporary or permanent effect.

The finding that cytotoxic effects after CBCT exposure were more prominent in younger patients, is an interesting observation. An explanation could be that pediatric epithelial tissues exhibit elevated rates of cellular proliferation and metabolic activity, rendering them more susceptible to cytotoxic effects [7, 40]. 

While children’s increased susceptibility to radiation-induced damage is well documented over the long term, immediate genotoxic effects measured under carefully optimized low-dose conditions may not differ significantly between children and adults. This finding aligns with previous research suggesting that at diagnostic radiation doses, especially when protocols are carefully optimized, the measurable differences in short-term genotoxic endpoints can be minimal [41–43].

Despite children being more sensitive overall, they may also have robust DNA repair mechanisms that help mitigate immediate damage. This compensatory response may yield comparable short-term genotoxic effects compared to adults, despite the long-term risk remains higher [44].

Moreover, tissue turnover and DNA repair capabilities exhibit substantial differences between children and adults. Pediatric epithelial tissues undergo more frequent renewal, heightening the probability of detectable cytotoxic events post-insult. Adult tissues, despite slower turnover, may exhibit reduced DNA repair capacity with age, potentially balancing out genotoxic outcomes among different age groups [45].

Because of the intricate interplay between the environment and genotype within the growth dynamics, development, and adaptability framework, it is challenging to understand the differences between researches on MN from a clinical standpoint. It is crucial to consider a few complicating variables in these kinds of investigations, such as viruses, immune system modifications, malfunctions in the DNA repair mechanism, etc. Age and different cell types can have an impact on cell growth [46]. 

The study’s findings on the genotoxic and cytotoxic effects of cone beam computed tomography (CBCT) raise significant concerns about its application, particularly among young patients, which aligns with established protocols from credible organizations.

The International Commission on Radiological Protection (ICRP) underscores caution in the application of dental CBCT for pediatric patients due to their heightened radiosensitivity and smaller size [47]. 

Dentomaxillofacial Paediatric Imaging: An Investigation into Low-Dose Radiation-Induced Risks (DIMITRA) has established patient-specific and indication-specific guidelines for the prudent use of CBCT in pediatric patients. The method promotes a shift from the principles of As Low As Reasonably Achievable (ALARA) and As Low As Diagnostically Acceptable (ALADA) to As Low As Diagnostically Acceptable, Indication-Oriented, and Patient-Specific (ALADAIP) [48]. 

## Conclusion

According to the current study, buccal mucosal cells may be subjected to cytotoxic and genotoxic effects from CBCT. While the genotoxic effects are still equal, the cytotoxic effects are more pronounced in the younger age group compared to adults. International guidelines should include stricter and more precise recommendations for pediatric patients on when CBCT is absolutely necessary for diagnostics. However, the use of CBCT imaging must be justified on a case-by-case basis due to the susceptibility of the pediatric patient to ionizing radiation. This study supports the need for careful, patient-specific justification for prescribing CBCT, particularly in pediatric cases, based on the measurable cytotoxic effects observed. This research is recommended on children with a bigger sample size and extended follow-up to evaluate the potential genotoxic effects of CBCT.

## Data Availability

No datasets were generated or analysed during the current study.

## Abbreviations

CBCT: Cone beam computed tomography
CT: Computed tomography
2D: Two-dimensional
3D: Three-dimensional
FOV: Field of view
MN: Micronucleus
DNA: Deoxyribonucleic acid
